# Aflibercept Nanoformulation Inhibits VEGF Expression in Ocular In Vitro Model: A Preliminary Report

**DOI:** 10.3390/biomedicines6030092

**Published:** 2018-09-11

**Authors:** Shannon J. Kelly, Anjali Hirani, Vishal Shahidadpury, Aum Solanki, Kathleen Halasz, Sheeba Varghese Gupta, Brian Madow, Vijaykumar Sutariya

**Affiliations:** 1 Department of Pharmaceutical Sciences, College of Pharmacy, University of South Florida, Tampa, FL 33612, USA; shannonk@health.usf.edu (S.J.K.); ahirani@gmail.com (A.H.); vpury89@gmail.com (V.S.); solankia@health.usf.edu (A.S.); halaszk@health.usf.edu (K.H.); svarghes@health.usf.edu (S.V.G.); 2 Department of Ophthalmology, College of Medicine, University of Florida, Jacksonville, FL 32209, USA; Brian.madow@jax.ufl.edu

**Keywords:** age-related macular degeneration, vascular endothelial growth factor, aflibercept, nanoparticles, PLGA

## Abstract

Age-related macular degeneration (AMD) is one of the leading causes of blindness in the United States, affecting approximately 11 million patients. AMD is caused primarily by an upregulation of vascular endothelial growth factor (VEGF). In recent years, aflibercept injections have been used to combat VEGF. However, this treatment requires frequent intravitreal injections, leading to low patient compliance and several adverse side effects including scarring, increased intraocular pressure, and retinal detachment. Polymeric nanoparticles have demonstrated the ability to deliver a sustained release of drug, thereby reducing the necessary injection frequency. Aflibercept (AFL) was encapsulated in poly lactic-co-glycolic acid (PLGA) nanoparticles (NPs) via double emulsion diffusion. Scanning electron microscopy showed the NPs were spherical and dynamic light scattering demonstrated that they were uniformly distributed (PDI < 1). The encapsulation efficiency and drug loading were 75.76% and 7.76% respectively. In vitro release studies showed a sustained release of drug; 75% of drug was released by the NPs in seven days compared to the full payload released in 24 h by the AFL solution. Future ocular in vivo studies are needed to confirm the biological effects of the NPs. Preliminary studies of the proposed aflibercept NPs demonstrated high encapsulation efficiency, a sustained drug release profile, and ideal physical characteristics for AMD treatment. This drug delivery system is an excellent candidate for further characterization using an ocular neovascularization in vivo model.

## 1. Introduction

A decrease in visual acuity can lead to alterations in the activities of daily living, quality of life, self-care, and mental health of an individual. A disease commonly responsible for this visual impairment is age-related macular degeneration (AMD). AMD is a degenerative disease of the macula and it affects the part of the retina responsible for sharp, detailed central vision. AMD is the most prominent cause of vision loss in people 50 years and older in North America and it is expected to affect 7.5 million people by 2020 in the United States [1]. Several factors influence the prognosis and progression of AMD, including genetic variation, living environment, and lifestyle [2].

Neovascular or “wet” AMD accounts for more than 90% of individuals with advanced vision loss. The distinctive feature of neovascular AMD is choroidal neovascularization (CNV), which is regulated by growth factors including vascular endothelial growth factor (VEGF) [1]. In CNV there is an abnormal proliferation of new blood vessels originating from the choroid, leading to sub- and intra-retinal macular edema, fluid leakage, bleeding, and fibrosis of retinal tissue. This results in severe visual deterioration [3].

Amongst the VEGF family of growth factors, VEGF-A is a signal protein that promotes angiogenesis and vascular leakage in the retina. VEGF’s biological properties arise from binding to trans-membrane VEGF receptors (VEGFR) on vascular endothelial cells. VEGF-A specifically binds to VEGFR-1 in the process of inflammatory neovascularization [2]. However, VEGFR-2 is responsible for triggering many of the proangiogenic functions of VEGF-A [4]. Drugs known as anti-VEGF agents have been designed to block this binding of VEGF, thereby limiting its damage to the eye. To date, the FDA has approved three anti-VEGF agents for ocular applications: Ranibizumab, pegaptanib, and aflibercept [5]. Aflibercept (AFL) is a fusion protein containing both VEGFR-1 and VEGFR-2, which binds to VEGF-A thus impeding its activity [2]. AFL’s prolonged duration of action is attributed to its distinctive binding activity. By blocking the VEGF signal with AFL, it has been shown there is stabilization and even reversal in the vision loss normally associated with neovascular AMD [3].

Many of the adverse effects of Anti-VEGF agents originate from their route of administration. Intravitreal injections are perhaps the most common mode of administration to the posterior segment. However, anti-VEGF agents possess a narrow therapeutic index, thereby necessitating frequent injections to maintain an acceptable concentration of drug in the eye [6]. This frequency not only reduces patient compliance, but also results in myriad potential adverse effects including retinal detachment, vitreous hemorrhage, and increased intraocular pressure [7,8]. Ocular protein delivery has the added challenges of short protein half-life and degradation by enzymes [9]. Polymeric nanoparticles deliver a targeted sustained release of drug, thereby reducing the injection frequency and related effects [10], as well as providing a therapeutic dose of protein with limited toxicity [11]. Furthermore, nanoparticles have demonstrated the ability to localize on the retinal pigment epithelium (RPE), an ideal trait for the amelioration of AMD [12].

Polymeric nanoparticles (NPs) can be prepared using various natural or synthetic materials. Poly lactic-co-glycolic acid (PLGA) is a biodegradable synthetic polymer which is commonly used for the preparation of NPs. The widespread use of PLGA in recent studies is partly due to its ability to demonstrate a sustained drug release with a low cytotoxicity and minimal side effects [13,14]. PLGA can be used to encapsulate hydrophobic or hydrophilic materials and the shape and size of PLGA NPs are easily manipulated [15]. For instance, Booysen et al. recently used PLGA to encapsulate vancomycin in a nanoformulation. The NPs were 247 nm but did not possess uniformity when analyzed under SEM. Regardless, the NPs were more effective in combating MRSA than a solution of free vancomycin [16]. Sanchez et al. used PLGA microspheres to encapsulate the macromolecule interferon-alpha (IFN-α). The sustained release and anti-proliferative activity of the microspheres indicate that this is a suitable in vitro platform for macromolecule delivery [11]. Feczko et al. studied the encapsulation of the protein bovine serum albumin (BSA) under various conditions. Using the double emulsion solvent evaporation method, PLGA nanoparticles encapsulating the protein were successfully produced with high encapsulation efficiencies [17]. Later, Swed et al. encapsulated lysozyme and human transforming growth factor beta 1 (TGF-β1) separately in PLGA NPs to examine protein encapsulation. They found that the NPs encapsulated at least 50% of each protein and possessed both a uniform size distribution (PDI less than 0.2) and sustained release properties over 30 days. Blank NPs were nontoxic to NIH3T3, HS68 and hATSC cells after 48 h; in fact the assay showed significant cell proliferation with increasing concentrations of NPs [18]. It may therefore be concluded that PLGA NPs are an appropriate mode of protein delivery.

There are several methods by which PLGA NPs can be produced. In this work, NPs containing AFL (AFL-NPs) were prepared via double emulsion diffusion using the synthetic biodegradable polymer PLGA. The NPs were then characterized for their physical characteristics, encapsulation efficiency, and sustained release profile. The cytotoxicity and efficacy of the NPs in human retinal pigment epithelial (ARPE-19) cells were determined via MTT assay and ELISA. By doing so, it was demonstrated that a high encapsulation efficiency, uniform size distribution and sustained drug release were achieved by the nanoformulation of the protein AFL.

## 2. Experimental Section

### 2.1. Materials

Aflibercept (Eylea, 2 mg/0.05 mL) (Regeneron Pharmaceuticals, Inc., Tarrytown, NY, USA) was generously donated by Dr. Brian Madow of the USF Eye Institute. ARPE-19 cells (ATCC^®^ CRL2302™) and DMEM: F-12 were ordered from the American Type Culture Collection (ATCC) (Manassas, VA, USA). 1× Dulbecco’s Modified Eagle’s Medium (DMEM) and 10× phosphate buffered saline (PBS) were purchased from Mediatech, Inc. (Manassas, VA, USA). PLGA-COOH (DL-lactide-co-glycolide) (PLGA) was obtained from LACTEL Absorbable Polymers (DURECT Corporation, Pelham, AL, USA). Dichloromethane (Chromasolv-HPLC grade), heat-inactivated fetal bovine serum, thiazoyl blue tetrazolium bromide (MTT reagent), and methanol (Chromasolv-HPLC grade) were purchased from Sigma-Aldrich (St. Louis, MO, USA). Polyvinyl alcohol (PVA) was purchased from Fisher Scientific (Pittsburgh, PA, USA). D-Mannitol was obtained from Acros Organics (Geel, Belgium). Slide-A-Lyzer Dialysis Cassettes (Extra Strength; 10,000 MWCO) were purchased from Thermo Scientific (Waltham, MA, USA).

### 2.2. Nanoparticle Preparation

**Figure 1:**
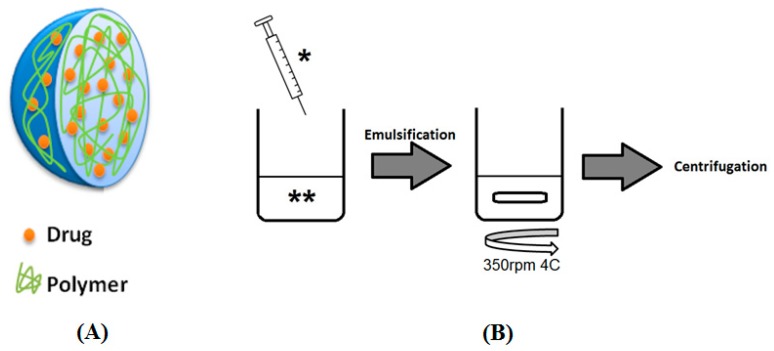
(**A**) Aflibercept nanoparticles were prepared via (**B**) the double emulsion diffusion method (* aqueous phase; ** organic phase).

The double-emulsion diffusion method was used to prepare AFL NPs (Figure 1). One milligram of AFL (25 µL stock solution) was added to 100 µL 1× PBS and a separate solution of 4.5 mg PLGA was prepared in 1 mL dichloromethane (DCM). These solutions were combined and emulsified using sonication at 9.5 W for approximately 30 s. After the dropwise addition of 2 mL 1% *w*/*v* PVA, the suspension was emulsified via sonication at 9.5 W for another 30 s. The emulsion was stirred at 500 rpm overnight at 4 °C to allow diffusion. The emulsion was split into two fractions and centrifuged at 20,000× *g* at 4 °C for 15 min. Each pellet was then resuspended in 1 mL 2% *w*/*v* mannitol. Blank NPs were prepared similarly excluding the addition of AFL.

### 2.3. Nanoparticle Characterization

The size and polydispersity index (PDI) of both the AFL and blank NPs were analyzed via dynamic light scattering (DLS) using a Wyatt DynaPro plate reader (Wyatt Technology Corporation, Santa Barbara, CA, USA). The NPs were diluted 1:200 in filtered deionized water to meet equipment specifications and analyzed in triplicate.

### 2.4. Scanning Electron Microscopy (SEM)

The NPs were viewed via SEM using a JOEL JSM-6490LV (JOEL Industries, Tokyo, Japan). Samples were diluted 1:10 in filtered deionized water and adhered to aluminum cylinders using a carbon polymer adhesive. All images were obtained using a 5 kV acceleration voltage.

### 2.5. Encapsulation Efficiency and Drug Loading

The encapsulation efficiency (EE) and drug loading (DL) of the NP formulation was calculated using the concentration of drug present in a 1 mL sample of the NPs. After centrifugation at 15,000× *g* for 5 min, the NPs were suspended in methanol and placed at 4 °C overnight to dissolve the NPs. Both this sample and a standard curve of AFL in methanol were prepared using the Pierce BCA protein assay (Thermo Scientific, Waltham, MA, USA) and analyzed via VIS spectroscopy at 562 nm on a BioTek Synergy H4 plate reader (BioTek Instruments Inc., Winooski, VT, USA). The standard curve was used to convert the absorbance of the NP sample to a concentration. EE and DL were calculated as follows:

%EE = (Mass of entrapped drug)/(Total mass of drug) × 100%

%DL = (Mass of entrapped drug)/(Mass of entrapped drug + Mass of polymer) × 100%.

### 2.6. In Vitro Release Studies

An in vitro release study was conducted according to a previously reported method [19]. Dialysis membrane cassettes were soaked in 1× PBS at 4 °C. Approximately 500 µL of AFL-NPs or AFL solution were inserted into the membrane and suspended in 100 mL PBS at 37 °C. Aliquots were removed and replaced with preheated PBS at regular intervals over a period of seven days. The collected samples were analyzed via BCA Protein Assay and read using spectroscopy at 562 nm with the Synergy H4 plate reader (Biotek Industries, Inc., Winooski, VT, USA). These values were compared to a calibration curve of AFL in 1× PBS to determine the cumulative percent of drug released at each interval.

### 2.7. Cell Culture

Human retinal pigment epithelial (ARPE-19) cells (ATCC^®^ CRL2302™) were grown in 1× Dulbecco’s Modified Eagle’s Medium (DMEM) with 10% *v*/*v* fetal bovine serum (FBS). Cells were kept in a Thermo Scientific Forma Steri-Cycle incubator (Fisher Scientific, Pittsburgh, PA, USA) at 37 °C and 5% carbon dioxide.

#### 2.7.1. Cytotoxicity

The cytotoxicity of the AFL-loaded and blank NPs in ARPE-19 cells was measured using the 3-(4,5-dimethylthiazol-2-yl)-2,5-diphenyltetrazolium bromide salt (MTT) assay [12]. ARPE-19 cells were seeded in a 48-well plate and incubated at 37 °C with 5% CO_2_ for 48 h to achieve confluence. Cell culture media was aspirated and the cells were treated with each of the NP formulations at final concentrations of 0.5 µM and 1 µM. After a 24-h incubation period, 300 µL of a 0.5 mg/mL solution of MTT reagent previously prepared in DMEM were added to each well. After another four hours of incubation, the MTT reagent was removed and 300 µL of DMSO were added to end the reaction. After shaking briefly, the plate was analyzed at 570 nm using a BioTek Synergy H4 plate reader (BioTek Instruments Inc., Winooski, VT, USA) to determine the relative amounts of live cells present in the sample. These values were reported as a percentage of the untreated control.

#### 2.7.2. VEGF-A Inhibition

ARPE-19 cells were seeded in a 48-well plate and incubated at 37 °C with 5% CO_2_ to achieve confluence and treated with each of the NP formulations at a final concentration of 0.5 µM. After 72 h, the expression of VEGF-A was quantified via enzyme-linked immunosorbent assay (ELISA) (Human VEGFA ELISA kit, Thermo Scientific, Waltham, MA, USA). Media samples were collected and analyzed using the Synergy H4 plate reader (Biotek Industries, Inc., Winooski, VT, USA) at 450 nm and 550 nm. VEGF-A expression was calculated as follows:

%Expression = (absorbance 450 nm − absorbance 550 nm)/(Control absorbance 450 nm − Control absorbance 550 nm) × 100%.

### 2.8. Statistical Analysis

Statistical analyses were carried out using GraphPad Prism 5, Version 5.02. Comparisons of each concentration of NP treatment, on both cell viability and VEGF-A expression, were completed using two-way ANOVA with Bonferroni post-tests to compare each treatment to the control, as well as other concentrations (if applicable). All results are written as mean values ± SD.

## 3. Results

### 3.1. Nanoparticle Characterization

**Figure 2:**
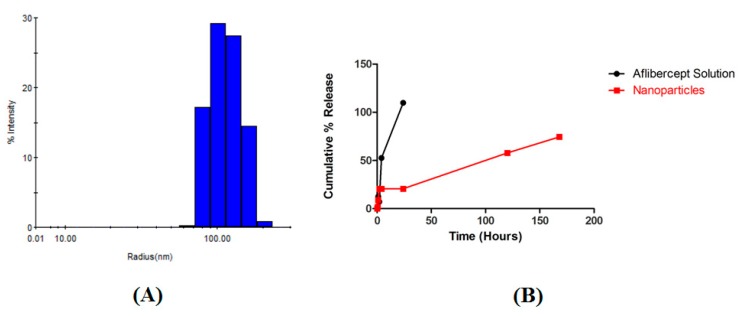
AFL-NPs were characterized for (**A**) size distribution and (**B**) in vitro release profile compared to AFL solution.

The diameter and PDI of the AFL and blank NPs were found in triplicate via DLS. AFL-NPs were larger than their respective blanks (Table 1). The PDI was consistently less than 1, indicating a uniform size distribution (Figure 2A).

### 3.2. Scanning Electron Microscopy (SEM)

**Figure 3:**
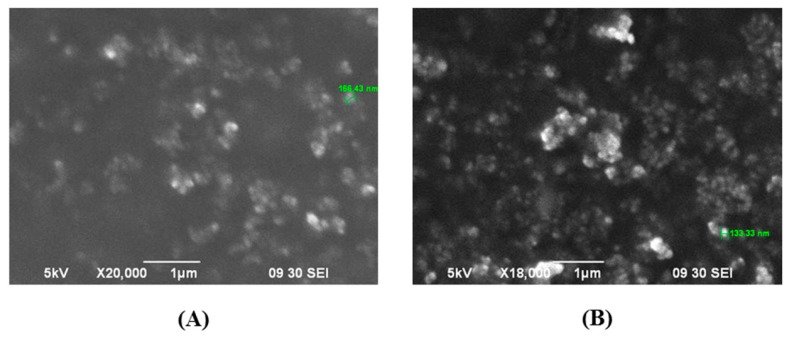
SEM micrographs of AFL-NPs were obtained at (**A**) 18,000× and (**B**) 20,000× magnification. Both images were obtained using 5 kV acceleration voltage.

AFL and blank NPs were visualized using SEM (Figure 3). Inspection showed that the NPs were spherical. The size and uniformity of the NPs corroborated with data determined via DLS.

### 3.3. Encapsulation Efficiency and Drug Loading

The EE and DL of each NP formulation was determined via VIS spectroscopy after analysis with the BCA protein assay. A standard curve of AFL in methanol was prepared to determine the concentration of AFL present in the NP fraction (r^2^ = 0.9247) (not shown). The NPs demonstrated an EE of 75.76 ± 2.59% and DL of 7.76 ± 0.24% (Table 1).

### 3.4. In Vitro Release

The rate of drug release from the NP formulation versus AFL in free solution was examined over seven days in 100 mL 1× PBS at 37 °C. AFL was fully released by solution within 24 h. Comparatively, the NPs released 74.49% in seven days. An initial burst release is demonstrated from the NPs in the first 2 h (Figure 2B).

### 3.5. Cytotoxicity

**Figure 4:**
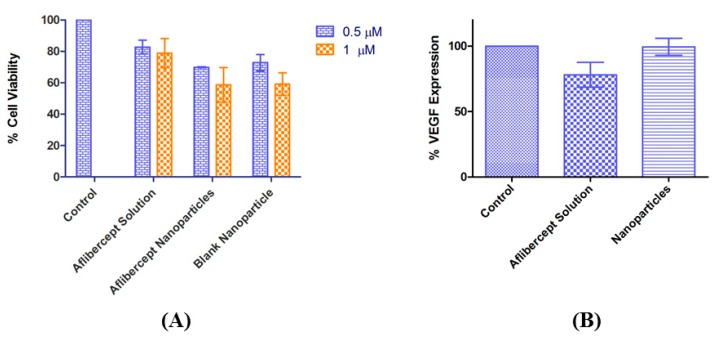
(**A**) AFL solution was non-toxic. AFL-NPs demonstrated similar cytotoxicity in ARPE-19 cells to blank NPs (*n* = 3, mean value ± SD); (**B**) after 72 h, VEGF-A expression by ARPE-19 cells was reduced post-treatment (0.5 µM) with AFL and AFL NPs (*n* = 3, mean ± SD).

The cytotoxicity of blank NPs, AFL NPs and AFL solution to ARPE-19 cells were determined via MTT assay. After 24 hours, 0.5 µM AFL NPs and blank NPs reduced cell viability in ARPE-19 cells by 30.11 ± 0.344% and 27.24 ± 5.22%, respectively compared to 17.29 ± 4.44% reduction caused by AFL. The 1 µM treatments of AFL NPs and blank NPs resulted in a 41.29 ± 10.99% and 40.87 ± 7.15% reduction in cell viability, and the AFL reduced viability by 21.16 ± 9.37%. The differences between the two concentrations of each treatment were considered insignificant (*p* < 0.05). Both NPs at each concentration showed significant toxicity compared to the control (*p* < 0.001). However, the AFL-loaded NPs showed no significant difference in cytotoxicity compared to the blank NPs (Figure 4A).

### 3.6. VEGF-A Inhibition

The effect of AFL and AFL NPs on the VEGF-A expression of ARPE-19 cells was examined via ELISA. After 72 h, VEGF-A was not significantly reduced by 0.5 µM AFL solution or AFL NPs. AFL solution reduced VEGF-A expression by 21.7 ± 16.4% and AFL NPs reduced expression by 0.6 ± 11.3% (Figure 4B). It must be noted that, though AFL solution is fully released into the surrounding media within 72 h, AFL NPs take over seven days to fully release (Figure 2B).

## 4. Discussion

The double emulsion diffusion method was used to prepare AFL NPs. Particle size data revealed that the drug-loaded NPs were 73 nm larger in diameter than similarly prepared blank NPs, confirming the presence of the drug. A NP of approximately 200 nm in diameter is ideal to localize to retinal pigment epithelial (RPE) cells; this is indeed demonstrated by the AFL NPs (Table 1). Additionally, the PDI of each formulation remained below one, demonstrating a uniform size distribution (Figure 2A). SEM micrographs showed that the NPs possessed a spherical morphology (Figure 3). Spherical NPs possess a high surface area to volume ratio compared to other shapes (rod, cube, etc.) resulting in a more reactive surface and ultimately more opportunities to produce a therapeutic effect [20]. Furthermore, the NPs demonstrated a Gaussian size distribution (0.2 PDI) thereby reducing any variability in this effect among the individual NPs (Figure 2A). Protein assay showed that the NPs encapsulated 75.76 ± 2.59% of the drug used in NP preparation and 7.76 ± 0.24% of the NP mass consisted of drug. As drug loading is a ratio of drug mass to NP total mass, this value may be increased by adding a higher ratio of drug to polymer during NP preparation. However, it has been previously noted that these changes have adverse effects on shape uniformity [21]. These results were consistently reproducible (data not shown) and corroborated with previously published studies [21,22,23].

Cytotoxicity studies demonstrated that free AFL is non-toxic. After 24 h, 0.5 µM and 1 µM concentrations of both AFL NPs and blank NPs significantly reduced cell viability in ARPE-19 cells (*p* < 0.001). However, there was no significant difference in the toxicity of the AFL NPs compared to blank NPs at each concentration (Figure 4A), meaning that this toxicity is due to the use of NPs rather than any inherent cytotoxicity of AFL. This may be due to the method of in vitro testing; if NPs settled to the flat bottom of the wells any cells trapped underneath would be suffocated. A similar scenario was observed by Irfan et al. as silica NPs produced sedimentation during an LDH assay [24]. They suggest that this is due to dose concentration and aggregation. Perhaps varying these factors would produce different results. The eye’s natural circulation prevents this adverse effect in vivo.

After 72 h, VEGF-A was significantly reduced in ARPE-19 cells by 0.5 µM AFL solution but not by the same concentration of AFL NPs (Figure 4B). It must be noted that, though AFL solution is fully released into the surrounding media within 72 h, AFL NPs take over seven days to fully release (Figure 2B). In vitro release studies showed that in 72 h, less than 57% of AFL was released into the surrounding media (Figure 2B). The sustained release properties of the NPs account for the low VEGF-A reduction by the AFL NPs. Hirani et al. reported a similar effect with triamcinolone acetonide. More potent inhibitory properties were demonstrated after 72 h compared to 12 h of treatment [12]. PLGA NPs formulated by Patel et al. illustrated a similar in vitro release profile with 25% of drug released after 48 h [25]. These sustained release properties allow the NPs to deliver a more powerful effect in vivo.

The current study demonstrates the potential of a novel nanoformulation containing AFL for the inhibition of VEGF in vitro. Further in vitro studies will be needed to demonstrate VEGF inhibition by the nanoformulation as well as AFL solution. Future in vivo studies will be carried out in a laser-induced choroidal neovascularization mouse model. The efficacy, toxicity, biodistribution and pharmacokinetics will be examined in this manner. Further clinical studies may be conducted in a human population to analyze the total effect of the nanoformulation. In summary, our nanoformulation of AFL shows potential as a protein drug delivery vehicle for the amelioration of ocular neovascularization in vitro.

## 5. Conclusions

Polymeric NPs were prepared for the sustained delivery of the protein aflibercept. The NPs were approximately 200 nm in diameter; an appropriate size for ocular therapeutics. The NPs were uniformly distributed and possessed a spherical morphology. The NPs demonstrated a sustained drug release over seven days and were not significantly more toxic to ARPE-19 cells than similarly prepared blank NPs. VEGF-A reduction in ARPE-19 cells was not significant after 72 h due to the sustained release property of the NPs. Future in vivo studies are needed to determine the effect of the NPs on live animal tissue as well as the clinical viability of the NPs in humans. Overall, aflibercept NPs demonstrated promising properties as a protein delivery vehicle for the future treatment of AMD and other neovascular conditions.

## Figures and Tables

**Table 1:** The characteristics of aflibercept NPs (*n* = 3, mean value ± SD).

NP Type	Diameter (nm)	PDI	%EE	%DL
Blank	169.91 ± 4.29	0.050 ± 0.077	-	-
AFL-NPs	243.13 ± 17.64	0.201 ± 0.071	75.76 ± 2.59	7.76 ± 0.24
